# Cell type-specific suppression of mechanosensitive genes by audible sound stimulation

**DOI:** 10.1371/journal.pone.0188764

**Published:** 2018-01-31

**Authors:** Masahiro Kumeta, Daiji Takahashi, Kunio Takeyasu, Shige H. Yoshimura

**Affiliations:** 1 Graduate School of Biostudies, Kyoto University, Kyoto, Japan; 2 Graduate School of Engineering, Kyoto University, Kyoto, Japan; 3 Center for Biotechnology, National Taiwan University, Taipei, Taiwan; UC San Diego, UNITED STATES

## Abstract

Audible sound is a ubiquitous environmental factor in nature that transmits oscillatory compressional pressure through the substances. To investigate the property of the sound as a mechanical stimulus for cells, an experimental system was set up using 94.0 dB sound which transmits approximately 10 mPa pressure to the cultured cells. Based on research on mechanotransduction and ultrasound effects on cells, gene responses to the audible sound stimulation were analyzed by varying several sound parameters: frequency, wave form, composition, and exposure time. Real-time quantitative PCR analyses revealed a distinct suppressive effect for several mechanosensitive and ultrasound-sensitive genes that were triggered by sounds. The effect was clearly observed in a wave form- and pressure level-specific manner, rather than the frequency, and persisted for several hours. At least two mechanisms are likely to be involved in this sound response: transcriptional control and RNA degradation. ST2 stromal cells and C2C12 myoblasts exhibited a robust response, whereas NIH3T3 cells were partially and NB2a neuroblastoma cells were completely insensitive, suggesting a cell type-specific response to sound. These findings reveal a cell-level systematic response to audible sound and uncover novel relationships between life and sound.

## Introduction

Many organisms are equipped with multiple environmental recognition systems that detect not only material, physico-chemical entities, but also many non-material factors, including heat, light, magnetic fields, gravity, pressure, and sound. As the smallest unit of life, cells also have systems for sensing non-material environmental factors. For example, heat induces specific responses mediated by heat-shock and cold-shock proteins (reviewed in[1–3]) and temperature-sensitive transcriptional regulators[4], light is recognized by light sensor molecules[5], and gravity and pressure are sensed by mechanosensitive molecules[6, 7]. All of these systems are mediated by molecular transducers that convert non-material information into electrical or biochemical signals leading to specific cellular responses. Specialized sensory cells, such as olfactory neurons, photoreceptor cells, and inner ear hair cells, that detect and transduce environmental information are well known and have been extensively investigated. However, little is known about the impact of audible sound on non-specialized cells and whether they are affected by the vibrational energy of sound waves.

Sound is a compressional mechanical wave that transmits oscillations of pressure through substances. Several investigations have utilized ultrasound, non-audible high-frequency (more than 20 kHz) sound, as a cellular stimulation[8–10]. There is accumulating evidence showing a positive effect of low-intensity pulsed ultrasound in bone repair by activating osteogenic activities (reviewed in[11]). Although the underlying mechanism is not fully understood, the ultrasound stimulation results in the upregulation of a set of osteoblastic differentiation genes including prostaglandin-endoperoxide synthase 2 (*Ptgs2*) (also known as cyclooxygenase-2, *COX-2*)[12, 13]. Analyses based on cultured cells have used stromal ST2 cells, which were derived from mouse bone marrow[14]. Stimulation with 1.5 MHz frequency ultrasound at a 1 kHz pulse frequency and an intensity of 30 mW/cm^2^ for 20 minutes upregulated the expression of osteoblastic differentiation genes in less than one hour[10, 15]. These results suggest that cellular mechanism(s) do respond to acoustic stimulation, but the lower range of sound frequencies remains unexplored.

Audible sound (20 Hz—20 kHz frequency) is virtually everywhere in nature, and one of the most important environmental information cues for animals. Humans perceive sound by coordinated processes including the auditory organ and the nervous system: perception at the eardrum as mechanical vibration and transmission through inner ear mechanics, conversion into the biochemical signal by inner ear hair cells, neuronal signal transmission to the brain, and finally integrated recognition in the auditory cortex. Because our auditory perception seems to be delegated to highly specialized cells, the potential of “generic” types of cells to directly recognize sound has not been investigated.

To understand whether there is a general cell-level perception of audible sound as an environmental factor, experiments with ST2 cells were designed to test a variety of audible sound parameters. In addition to the above-mentioned ultrasound-sensitive genes, expression levels of genes related to mechanotransduction (reviewed in[6, 16, 17]), including connective tissue growth factor (*CTGF*) (also known as CCN family member 2, *CCN2*)[18] and an extracellular matrix protein Tenascin-C (*TNC*)[19], were also analyzed by real-time quantitative PCR. Experiments using conditional sound treatment, drug treatment, and different cell lines were also performed to obtain further biological insights into the cellular response to audible sound.

## Materials and methods

### Cell culture

ST2, NIH3T3, C2C12, and NB2a cells were provided by the RIKEN BRC through the National Bio-Resource Project of the MEXT, Japan. The cells were cultured, following the provider’s recommendations, in a water jacketed incubator supplied with 5% CO_2_ (SANYO/Panasonic, MCO-175). Standard ϕ60 or ϕ100 mm dish was used for sound exposure experiments. To avoid the effect of contact inhibition and spontaneous differentiation triggered by confluent culture, the cells were carefully cultured under 60% confluence throughout the experiments. Cells were placed in the incubator with speaker for at least overnight to stabilize the condition before sound exposure. α-Amanitin (Sigma, A2263) was added at 5 μM when needed. For the water block assay, ϕ60 mm dish was placed in a water bath. A wire support was used to avoid movement of the dish during experiment.

### Sound materials and systems

The sounds used in this study were: 440 Hz sine-wave, 440 Hz triangle-wave, 440 Hz square-wave, 55 Hz sine-wave, 110 Hz sine-wave, 4 kHz sine-wave, and white noise, all generated by NCH Tone Generator software (NCH Software) **(S1–S7 Files)**. The wave form and the averaged spectrum were analyzed by SP4Win custom software (NTT). A full-range speaker was used for the assay (Fostex, 6301NB). A sound level meter (RION, NL-31) was used to monitor the sound pressure level (SPL) alongside the cell culture dish. Four different SPLs, 94.0, 88.0, 82.0, and 76.0 dB, were used in this study, which correspond to 1, 0.5, 0.25, and 0.125 Pa pressures, resepectively.

Transmission loss (TL) of the sound energy at the polystyrene lid of the dish was estimated by the mass law: TL = 20 log_10_ f×m—43 dB, where f represents frequency and m represents area density. In our experimental condition using 440 Hz sound, TL was estimated to be 10.1 dB. Therefore, when emitting 94.0 dB sound from the speaker, approximately 83.9 dB sound will reach inside the dish through the lid, which corresponds to 0.313 Pa pressure. Transmittance of the sound energy from air to water (E_a-w_) is calculated as E_a-w_ = 1—R^2^, where R represents an acoustic reflection coefficient, which is estimated by R = (ρ_w_×V_w_—ρ_a_×V_a_) / (ρ_w_×V_w_ + ρ_a_×V_a_) (ρ_w_ and ρ_a_ represents densities of water and air, and V_w_ and V_a_ represents sound velocity in water and air, respectively). Given the standard values of densities and velocities of air and water (ρ_w_: 997.1 kg/m^3^, ρ_a_: 1.184 kg/m^3^, V_w_: 1497 m/s, V_a_: 346.5 m/s at 25°C), E_a-w_ value will be about 1.10×10^−3^. SPL of the sound transmitting from air to the water can then be estimated by *p*_w_^2^ = 1.10×10^−3^ *p*_a_^2^, (*p*_w_ and *p*_a_ represents sound pressure in water and air, respectively). Overall, when emitting 94.0, 88.0, 82.0, and 76.0 dB sound from the speaker, the sound pressure directly transmit to the culture medium will be about 10.4, 5.2, 2.6 and 1.3 mPa, respectively, in our experimental system.

### RNA extraction and real-time quantitative PCR analysis

Total RNA extraction was performed with an RNeasy kit (Qiagen, 74104). The extracted total RNA was directly used as a template for RT-qPCR using a One Step SYBR PrimeScript Plus RT-PCR kit (Takara, RR096B), and an RT-qPCR system (Roche, Light Cycler 480). Quality of the probes was verified by using different amounts of total RNA **(S1 Fig)**. Melting curve analysis was performed to check the quality of the amplicon, and only the single-peaked runs were selected. The standard line of crossing points for each gene was used to quantify each data. The amounts of loading templates in each conditions were calibrated by the *ACTB* mRNA level, and values relative to the mRNA levels from unexposed, pre-sound samples were obtained. All the assays in this study were performed in the detection range of templates showing a linear relationship to the crossing point. Statistical significance was judged for each gene by comparing mRNA levels of time 0 (pre-sound) with each time point after sound exposure by t-test.

Throughout the experiments, the confluency of cells was carefully kept below 60% to avoid spontaneous differentiation induced by confluent culture, which can be monitored by the increase of osteoblastic differentiation markers osteoprotegerin (*OPG*) and collagen type I alpha 1 (*COL1a1*) genes **(S2 Fig)**.

## Results

### ST2 cells respond to sine-wave single frequency sound

**Fig 1:**
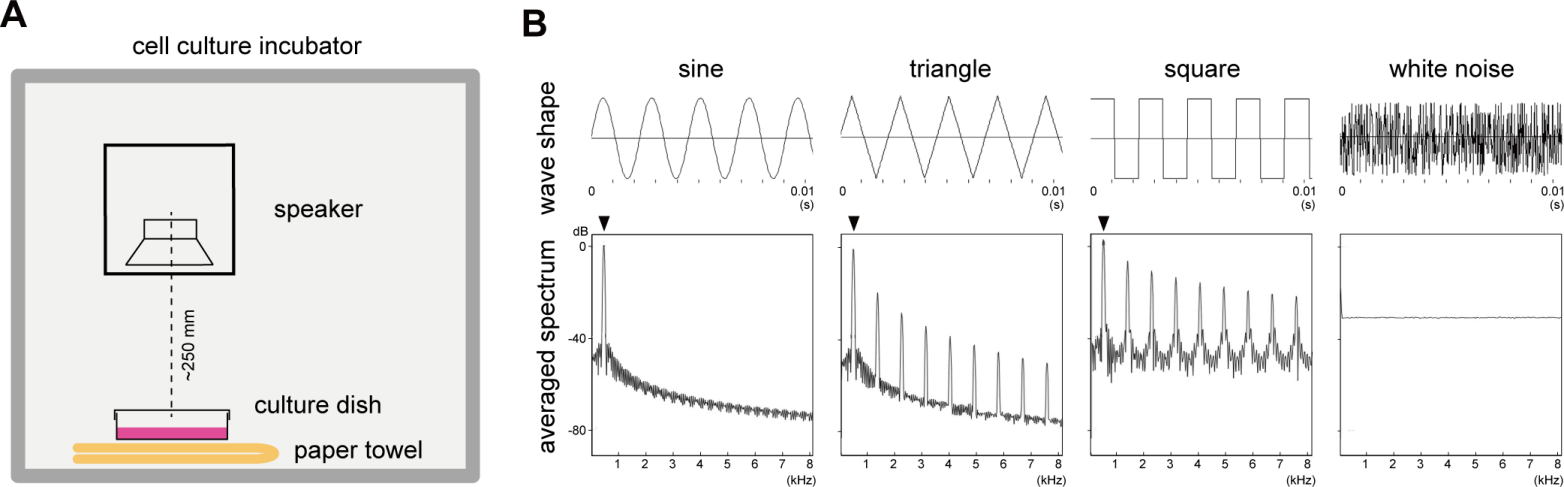
Experimental setup and the sounds used in the experiment. (A) A full-range speaker was located in a cell culture incubator. The speaker body was tilted to direct the speaker output straight toward the cell culture dish. (B) Shape and averaged spectrum of the sound used in this assay. 440 Hz sine-, triangle-, and square-waves were used as well as white noise, the mixture of all frequencies of sounds. Displacement in 0.01 second (upper panels) and averaged spectrum of the frequency composition up to 8 kHz (lower panels) are shown. 440 Hz positions are indicated by arrowheads.

A tunable full-range loud speaker was placed in a cell culture incubator and several different forms of audible sounds were emitted **(Fig 1A)**. The sounds used in this study were generated by NCH Tone Generator software **(S1–S7 Files)**. The wave forms and spectra were analyzed by SP4Win Custom software **(Fig 1B)**, and the sound pressure level (SPL) was monitored with a sound-level meter placed alongside the cell culture dishes. The background SPL in the incubator was measured to be 62–65 dB. ST2 cells cultured in ϕ60 or ϕ100 mm dishes were exposed to the sound with 94.0 dB SPL, which corresponds to 1.00 Pa and was the loudest sound emitted by our system without clipping noise. Total RNA was extracted over a time course and subjected to real-time quantitative PCR (RT-qPCR) analysis. Genes related to osteoblastic differentiation (*Ptgs2*) and mechanotransduction (*CTGF*, *TNC*) were analyzed along with reference control genes (*GAPDH*: glyceraldehyde-3-phosphate dehydrogenase, *ACTB*: β actin), using gene-specific primer sets **(S1 Fig)**. Relative mRNA amounts were determined using the *ACTB* mRNA level as a reference.

**Fig 2:**
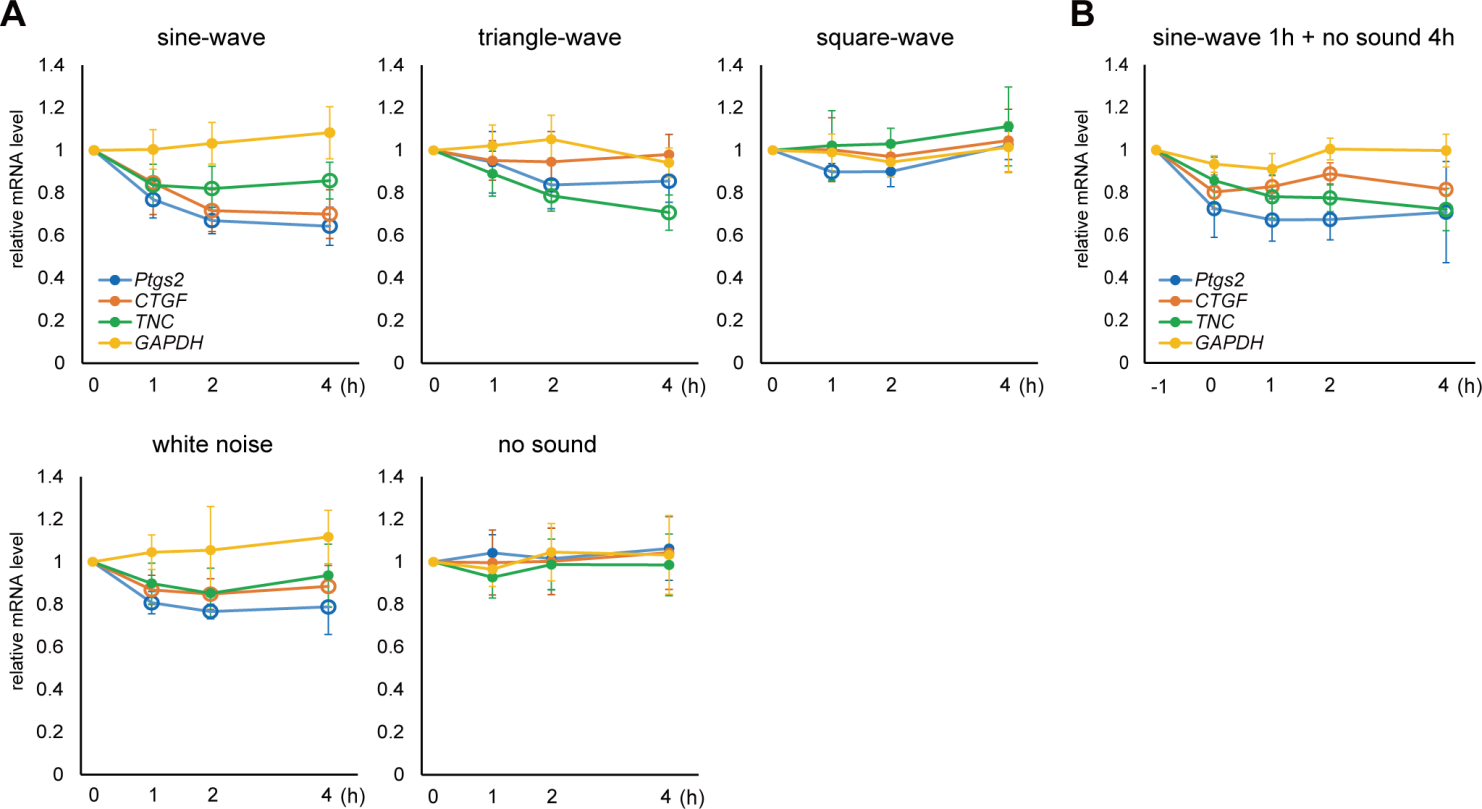
The effect of different sound on target genes and the persistency of the effect. (A) ST2 cells were exposed to 440 Hz, 94.0 dB, sine-, triangle-, and square-wave sound, and 94.0 dB white noise. Total RNA was extracted at different time points (0, 1, 2, 4 hours) and the amount of mRNAs for *Ptgs2*, *CTGF*, *TNC*, *GAPDH*, and *ACTB* were analyzed by RT-qPCR. The *ACTB* mRNA level was used as a reference to normalize the samples (see Methods and Methods for quantification). Relative mRNA levels compared to the pre-treated sample (0 hour) are shown. Open circles indicate statistically significant changes of each gene at each time point compared with 0 hour (P < 0.01). (Error bars represent standard deviation, n > 7 for sine-wave and no sound, and n > 5 for triangle-wave, square-wave, and white noise.) (B) ST2 cells were exposed to 440 Hz, 94.0 dB sine-wave sound for 1 hour (from -1 to 0 hour in the graph), after which the sound was stopped and mRNA levels were monitored by total RNA extraction and RT-qPCR over a time course of 0, 1, 2, and 4 hours. Relative mRNA amounts were shown. Open circles indicate statistically significant changes of each gene at each time point compared with 0 hour (P < 0.01). (Error bars represent standard deviation, n > 5.).

When using sine-wave sound, expression levels of *Ptgs2*, *CTGF*, and *TNC* genes were significantly downregulated to 80–60% in 1–2 hours **(Fig 2A, sine-wave)**. Relative mRNA level of *GAPDH* remained unchanged, indicating significant suppression of those genes compared to control genes. A prominent effect was observed in the *Ptgs2* and *CTGF* genes, which showed an approximately 30% decrease in the respective mRNA levels in one hour, whereas *TNC* exhibited a slightly less but significant suppression. Reproducibility of the result was ensured by repeating the independent experiments for 3 times, for both sin-wave and no sound control, each accompanies RT-qPCR analysis with triplicated measurements. This suppression was not the indirect effect of the changes in differentiation status, since the differentiation marker genes *OPG* and *COL1a1* were not significantly changed by the same sound stimulus **(S2 Fig)**. Thus, the observed gene responses were actually triggered by the sound stimulation, not by the on-going differentiation which accompanies global alteration in gene expression.

### Effects of different wave forms and frequencies, and persistency of the effect

In addition to sine-wave sound, cells were exposed to triangle- and square-wave sounds with the same frequency and SPL (440 Hz and 94.0 dB, respectively). Interestingly, these sounds had less of a suppressive effect on the target gene expressions. *TNC* showed significant suppression by triangle-wave, while the response of *Ptgs2* or *CTGF* were not as obvious as sine-wave sound **(Fig 2A, triangle-wave)**. More interestingly, square-wave sound had almost no significant effect on any of the genes tested **(Fig 2A, square-wave)**, demonstrating that the wave form is an important factor for sound-induced gene regulation. White noise, a mixture of all frequencies, also showed significant suppression of the target genes **(Fig 2A, white noise)**. Persistence of the gene response was then analyzed. The cells were exposed to sine-wave sound (440 Hz and 94.0 dB) for only one hour, and then cellular RNA was analyzed at different time points during the silent chase period. The results revealed that once suppressed by the sound, the target mRNA level remained low for at least 4 hours **(Fig 2B)**.

**Fig 3:**
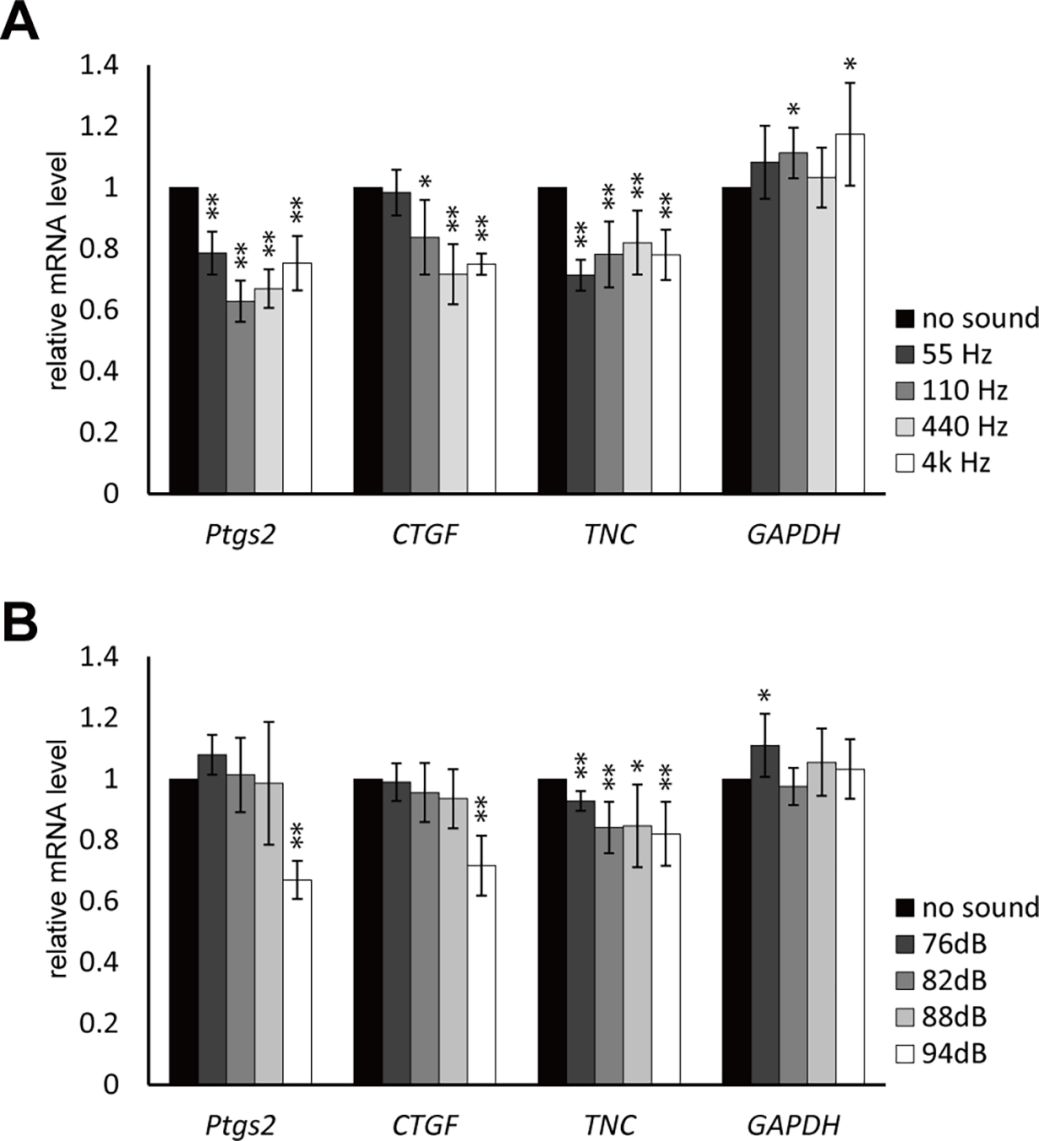
The effect of different frequency and sound pressure level on the target gene regulation. (A) ST2 cells were exposed to 94.0 dB sine-wave sound with 55 Hz, 110 Hz, 440 Hz, and 4 kHz frequencies. Total RNA was extracted 0 and 2 hours after the sound exposure. (Error bars represent standard deviation, n = 3. * indicates P < 0.05 and ** indicates P < 0.01 to the 0 hour sample.) (B) ST2 cells were exposed to 440 Hz sine-wave sound at 76.0, 82.0, 88.0, and 94.0 dB SPL. Total RNA was extracted 0 and 2 hours after the sound exposure. (Error bars represent standard deviation, n = 6. * indicates P < 0.05 and ** indicates P < 0.01 to the 0 hour sample.).

Since maximum suppression was observed within 2 hours in most cases, standard sound exposure time was set as 2 hours to further investigate the effect of frequency and SPL. In contrast to the wave form, the frequency had little effect; sine-waves with different frequencies (55, 110, 4k Hz) suppressed three genes similarly **(Fig 3A)**. The effect of the pressure level was analyzed using 88.0, 82.0, and 76.0 dB outputs, which correspond to 0.50, 0.25, and 0.125 Pa, respectively. *Ptgs2* and *CTGF* showed clear correlation to the loudness; almost no effect was observed in 88.0, 82.0, and 76.0 dB **(Fig 3B)**. However, *TNC* exhibited significant suppression in all different loudness tested, suggesting a different working mechanism of sound stimulation to this gene.

### Physical and cellular mechanisms of sound-evoked suppression of gene expression

**Fig 4:**
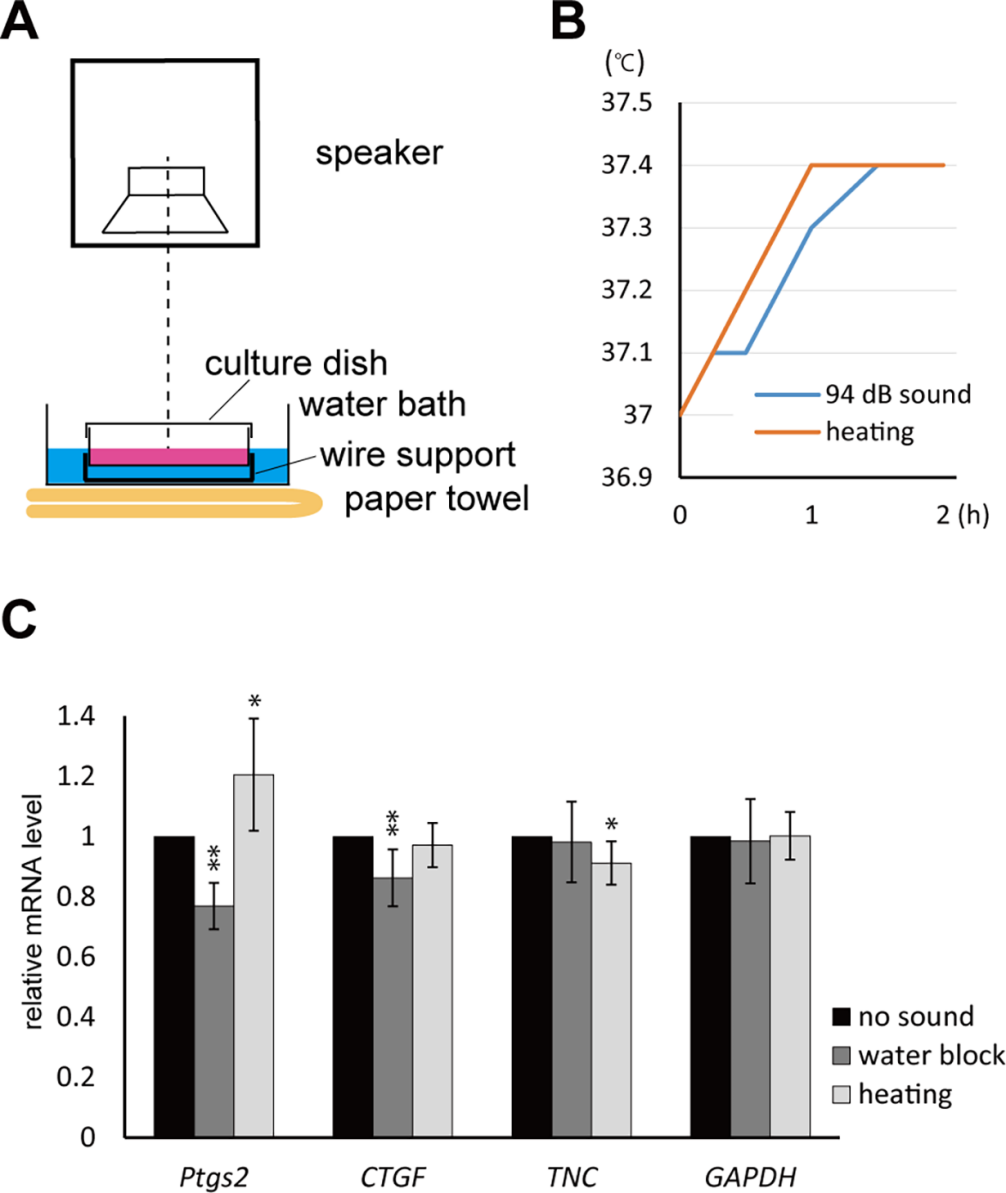
Water block and heating assays. (A) Water block assay was performed by floating a culture dish on a water bath in the incubator. Wire support was used to avoid movement of the dish during the assay. (B) Emission of 440 Hz, 94.0 dB, sine-wave sound resulted in the 0.4°C increase in the temperature of the culture medium in a dish (blue line). The effect of the heat itself was tested by changing temperature setting of the incubator to mimic similar heating condition (orange line). (C) Summary of the RT-qPCR result of water block (with 440 Hz, 94.0 dB, sine-wave sound) and heating (without sound) conditions. Total RNA was extracted 0 and 2 hours. (Error bars represent standard deviation, n > 5. * indicates P < 0.05 and ** indicates P < 0.01 to the 0 hour sample.).

Apart from the direct transmission of compressive wave from air to the cells in culture medium, there are several possible effect caused by the sound emission. One possibility is the radiated compressive waves due to sound-induced transverse vibration of the dish. To investigate the effect of the limited path, from air to water of compressive sound waves, water block condition was tested by floating the whole dish in a water bath in the cell culture incubator **(Fig 4A)**. Another possibility is the heating effect. The 94.0 dB sound emission caused 0.4°C increase of the temperature of the medium in the dish **(Fig 4B)**. This effect of heat was also tested by changing temperature setting of the incubator to follow similar temperature change without sound. The RT-qPCR results showed significant suppression of *Ptgs2* and *CTGF* by the sound in the water block condition **(Fig 4C, water block)**. These two genes were not suppressed by the heat alone without sound; *Ptgs2* gene expression was rather enhanced **(Fig 4C, heating)**, suggesting that they are suppressed in response to the sound wave transmitting from air to the medium. By contrast, the suppression of *TNC* gene was not observed in water block condition, but observed slightly by the heat **(Fig 4C)**. This suggests that the suppression of *TNC* might be caused by the factor(s) other than the compressional sound pressure.

**Fig 5:**
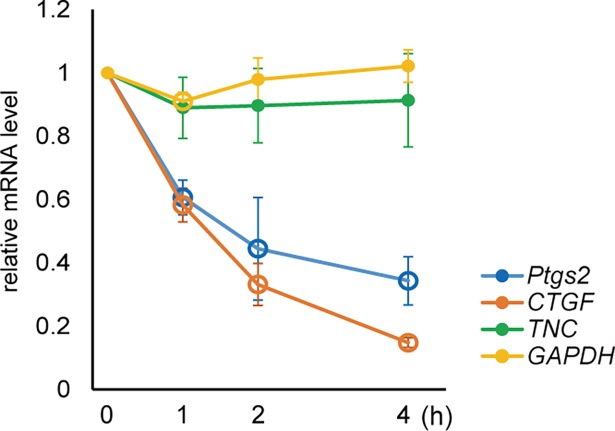
Sound-triggered gene response in the presence of RNA polymerase II inhibitor. ST2 cells were pre-treated with 5 μM α-amanitin for 1 hour and subjected to 440 Hz, 94.0 dB sine-wave sound for 0, 1, 2, and 4 hours. Open circles indicate statistically significant changes of each gene at each time point compared with 0 hour (P < 0.01). (Error bars represent standard deviation, n > 5.).

We then examined whether the apparent decrease in the *Ptgs2*, *CTGF*, and *TNC* mRNA levels was due to the suppression of RNA synthesis or activation of RNA decay. To test if the suppression is coupled with the mRNA transcriptional activity, cells were treated with α-amanitin, an inhibitor of RNA polymerase II, one hour prior to the sound stimulation. In this condition, the relative decrease of *Ptgs2* and *CTGF* mRNA levels was still observed and rather enhanced **(Fig 5)**. This means that the suppression of these mRNAs occurred in a transcription-independent manner, possible by activating decay of them. By contrast, the sound-dependent suppression of *TNC* was not significant in the α-amanitin treated condition, suggesting that the sound-dependent suppression of *TNC* gene is coupled with transcriptional activity. These results imply the existence of at least two different mechanisms for gene suppression by the sound stimulation.

### Variations of the sound-dependent gene regulation system among cell types

**Fig 6:**
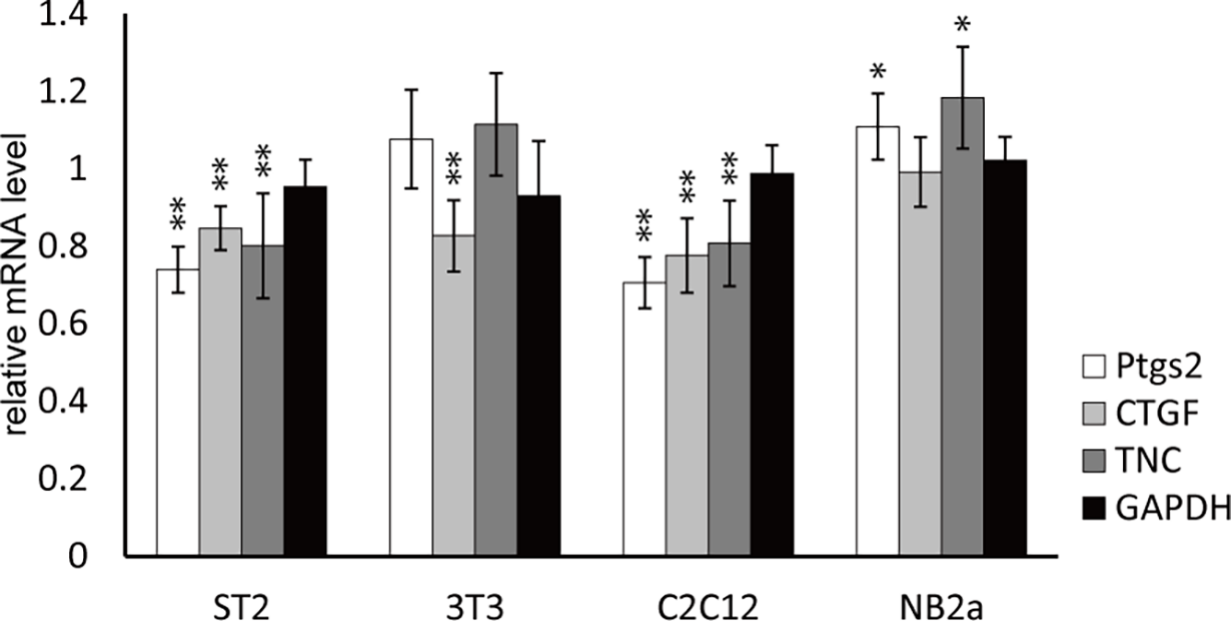
The effect of sound on different cell types. ST2, NIH3T3, C2C12, and NB2a cells were subjected to 440 Hz, 94.0 dB sine-wave sound for 1 hour. mRNA levels of *Ptgs2*, *CTGF*, *TNC* were analyzed along with *GAPDH* and *ACTB* reference genes. (Error bars represent standard deviation, n > 5. * indicates P < 0.05 and ** indicates P < 0.01 to the 0 hour sample.).

In addition to the ST2 stromal cells, NIH3T3 fibroblasts, C2C12 myoblasts, and NB2a neuroblastoma cells were subjected to the sound stimulation, using 440 Hz sine-wave sound with 94.0 dB SPL. The result revealed significant variations of the responses among different cell types **(Fig 6)**. C2C12 cells exhibited similar responses to ST2 cells, showing an apparent suppression of all three genes. On the other hand, the *Ptgs2* and *TNC* genes were not sensitive to sound in 3T3 cells, and none of the three genes were suppressed by sound in NB2a cells. These results suggest that sound-triggered gene regulation is shared among several different cell types, but differs slightly, depending on individual cellular physical properties and/or subcellular systems.

## Discussion

In this report, we have shown alterations in the expression levels of representative genes that were triggered by audible sound stimulation. Characteristic features of the sound-sensitive gene regulation revealed in this study are: 1) downregulation of the target genes; 2) rapid and persistent effect; and 3) wave-form dependency.

The property of the audible sound is vastly different from mechanical stresses used in mechanobiological studies, and also from ultrasound, both of which have been known to change gene expression patterns. For example, one of the well-studied mechanical stimuli is blood flow, where mechanical fluid stress is crucial for the development and maintenance of blood vessels (reviewed in[20]). Compared to the periodic pressure of the blood flow (10–20 kPa and ~1 Hz in humans), audible sound provides far less pressure (about 1 Pa at 94.0 dB) and much higher frequency (20 Hz—20 kHz for audible sound). Therefore, sound could be a stimulus which is distinct from other well-studied repetitive mechanical stimuli, and therefore may induce unique cellular responses. The sound pressure transmits to the cells in our experimental set up was estimated to be 10.4 mPa at 94.0 dB output (see Materials and Methods for estimation). Although this is quite a small perturbation compared to other mechanical stimuli, this may be sensible enough for mechanosensitive molecular machineries such as α-catenin, which recognizes several hundred pN range of tension forces and transmit signals[21].

Mechanotransduction pathways include many different types of molecular mechanisms, such as small- and trimeric-G proteins, kinases and phosphatases, lipid/calcium metabolism, and cytoskeletal elements, finally leading to the reorganization of cellular activities[22–25] and adaptive gene regulation (reviewed in[7, 26–29]). Dynamic 15% compressive strain of chondrocytes was shown to inhibit IL-1β induced upregulation of *Ptgs2* gene[30]. Such mechanical forces induces chromatin condensation through histone modifications, leading to mark the long-term persisting “mechanical memory” to the nucleus[31]. Since the sound stimulation showed similar suppressive and persistent effect on *Ptgs2* gene, it is reasonable to suggest that cellular sound sensing share the same mechanism with the compressional force transmission pathway. Another type of the mechanical stimuli, fluid flow stress, is known to upregulate *CTGF* gene expression in an intensity-dependent manner[32]. Interestingly, after applying mechanical stress to cells, relaxation from the stress lowered the *CTGF* mRNA level to less than 25% in 6 hours[18]. Taken together with these findings, the apparent down-regulation of *CTGF* gene by sound suggest that sound might act to remove the mechanical stress from the cells, thus attenuating the mechanical stress-inducible genes.

Contribution of the cytoskeleton-dependent pathway is also suggested. Mechanical stimulation applied to the plasma membrane by a magnetic particle resulted in the reorganization of the actin cytoskeleton, nuclear translocation of an actin-related transcription factor, megakaryoblastic acute leukemia factor-1 (MKL), and induced chromatin remodeling[33]. This pathway is quickly activated by the stimuli and induces an accumulation of MKL in the nucleus within a few minutes. *TNC* is known as one of the MKL-targeted genes, of which mRNA level was two-fold upregulated by cyclic tensile strain (10%, 0.3 Hz) for 6 hours in a MKL-dependent manner[19, 34]. Considering the analogy of acoustic waves converting their energy into the vibration of strings, it seems plausible that there could be a similar involvement in the cytoskeleton-mediated gene regulation pathway, either direct force transmission system or signaling molecule-dependent control.

Ultrasound is widely known and used to induce osteoblastic differentiation both *in vitro*[10, 13, 15, 35] and *in vivo*[8, 9]. The mechanisms elucidated so far include membrane perturbation[36], organellar displacement[37], intracellular cavitation[38], and cytoskeletal reorganization[39]. The resulting gene regulation occurs rapidly and persists for a long time; typically 20–40 minutes ultrasound treatment induces detectable gene responses within 1 hour, and persists for at least 24 hours[10, 15, 40]. This is a similar timeframe to sound-induced gene response, and suggests the involvement of the similar molecular mechanisms. In addition, it has been reported that ultrasound affects molecular behaviors: enhancing molecular diffusion in agarose gels[41] and increasing supercoiling of plasmid DNA[42]. Although the frequency and energy of audible sound is vastly different from those of ultrasound, both cause frequent perturbations of substances and thus their effect on cells may be explained at the molecular level.

We have demonstrated that RNA decay is one of the potential pathways affected by audible sound stimulation **(Fig 5)**. Both *Ptgs2* and *CTGF* mRNAs contain AU-rich elements (ARE) at the 3’ untranslated region, which function as an active degradation signal[43, 44]. ARE-containing mRNAs are targeted to processing bodies for degradation[45, 46], and/or stress granules under stressed conditions such as oxidization and proteasomal inhibition[47, 48]. Taken together with the notion that acoustic waves can oscillate organellar-level subcellular structures[37], sound may directly affect the ARE-dependent mRNA degradation pathway by vibrating the mRNA-containing subcellular domains.

Sensitivity to the sound stimulation was different in different cell types, which may be correlated with their differentiation ability. Among 4 different cell types tested in this study, the highest sensitivity was found in ST2 stromal cells and C2C12 myoblasts **(Fig 6)**. ST2 cells differentiate into osteoblasts and C2C12 cells differentiate into myotubes or osteoblasts depending on their environment[49]. In contrast, NB2a neuroblastoma, which has almost terminally differentiated, showed only a limited sound response. Undifferentiated cells are known to possess “open” chromatin structures with less heterochromatin regions, that change into “closed” status during differentiation by epigenetic regulations[50, 51]. Since undifferentiated cells in culture are expected to exist under transition among multiple differentiation pathways, it is reasonably speculated that sound perturbation acts more on such fluctuating status of cells by affecting chromatin structures. Cell type-specific response is also important to understand the significance of sound perception system in cell under physiological/pathological conditions. Recently a single nucleotide polymorphism on *IKBKAP* gene was identified as a cause of familial dysautonomia, which results in the decrease in the IKBKAP protein level by inducing abnormal mRNA splicing[52]. Since dysautonomia is one of the major noise-induced pathology, sound-triggered cell-level gene response at specific neuronal cells may be one of the causes for pathological disorders.

In our limited study, we have found only downregulation of genes by audible sound. More comprehensive studies may reveal upregulated genes, as many other stress response and environmental sensing systems consist of both up- and down-regulation of related genes. As a case in point, it is conceivable that NB2a cells will yet show a tissue-specific altered transcriptional profile. Considering the rapid response of the sound-triggered gene suppression and the non-invasive, harmless property of sound, manipulation of genes by sound (sonogenetics) may open up opportunities for biological use in the future. Further studies investigating other aspects of cellular activities in addition to the gene response will reveal how cells orchestrate their activities in response to sound, and uncover novel relationships between life and sound (sonobiology).

## Supporting information

S1 Fig Property of the qPCR probes. (A) Sequence of the probes designed for mouse target genes. (B) Crossing points of the RT-qPCR for each probe sets using different amounts of template. All the assays in this study were performed in the detection range of templates showing a linear relationship to the crossing point. (C) Calculated efficiency and correlation coefficient of the probe sets.
(TIF)

S2 Fig Result of RT-qPCR using high/low confluent ST2 cells. ST2 cells undergo spontaneous differentiation to osteoblast-like status in confluent culture conditions. High confluent culture spontaneously induces osteoblastic differentiation, which could be monitored by the significant increase in the *OPG* and *COL1a1* mRNA levels (upper graph). This induction was not observed in low confluent status (middle graph). Sound emission did not change the expression levels of differentiation marker genes at low confluent status within 4 hours (lower graph). All the experiments in this study were performed below 60% confluence to exclude the effect of gene regulation accompanied by differentiation, by monitoring *OPG* or *COL1a1* expression levels.
(TIF)

S1 Table A table of crossing point (Cp) values obtained by RT-qPCR. (XLSX)

S1 File Sound data 1. 440 Hz sine-wave sound (60 sec).
(WAV)

S2 File Sound data 2. 440 Hz square-wave sound (60 sec).
(WAV)

S3 File Sound data 3. 440 Hz triangle-wave sound (60 sec).
(WAV)

S4 File Sound data 4. 55 Hz sine-wave sound (60 sec).
(WAV)

S5 File Sound data 5. 110 Hz sine-wave sound (60 sec).
(WAV)

S6 File Sound data 6. 4 kHz sine-wave sound (60 sec).
(WAV)

S7 File Sound data 7. White noise (60 sec).
(WAV)
