# Supplementary figures and images for: Cell type-specific suppression of mechanosensitive genes by audible sound stimulation
Source: PLoS One. 2018 Jan 31;13(1):e0188764. doi: 10.1371/journal.pone.0188764 (PMC5791945; doi:10.1371/journal.pone.0188764)

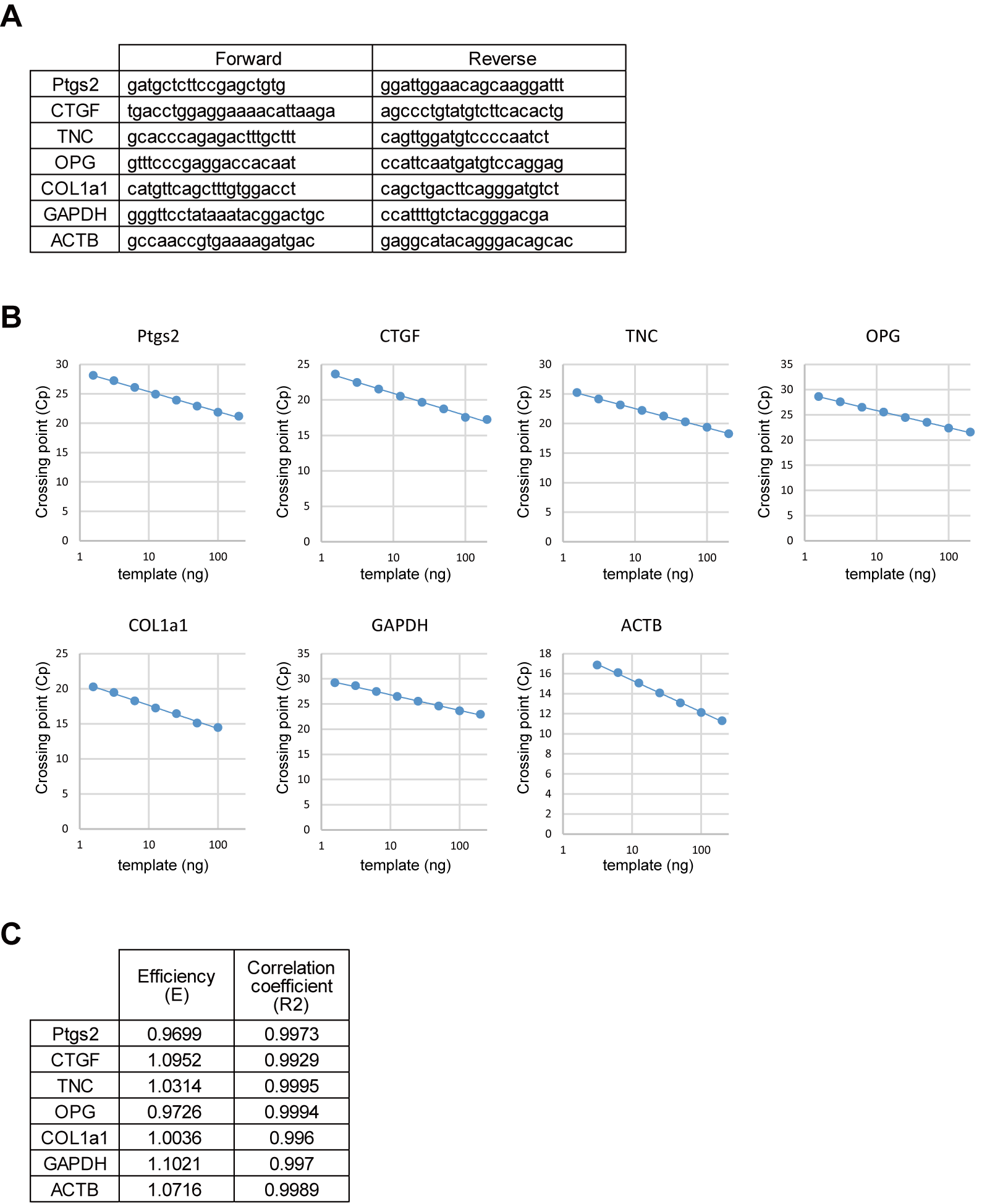

Supplement: S1 Fig — (A) Sequence of the probes designed for mouse target genes. (B) Crossing points of the RT-qPCR for each probe sets using different amounts of template. All the assays in this study were performed in the detection range of templates showing a linear relationship to the crossing point. (C) Calculated efficiency and correlation coefficient of the probe sets. (TIF) [file pone.0188764.s001.tif]

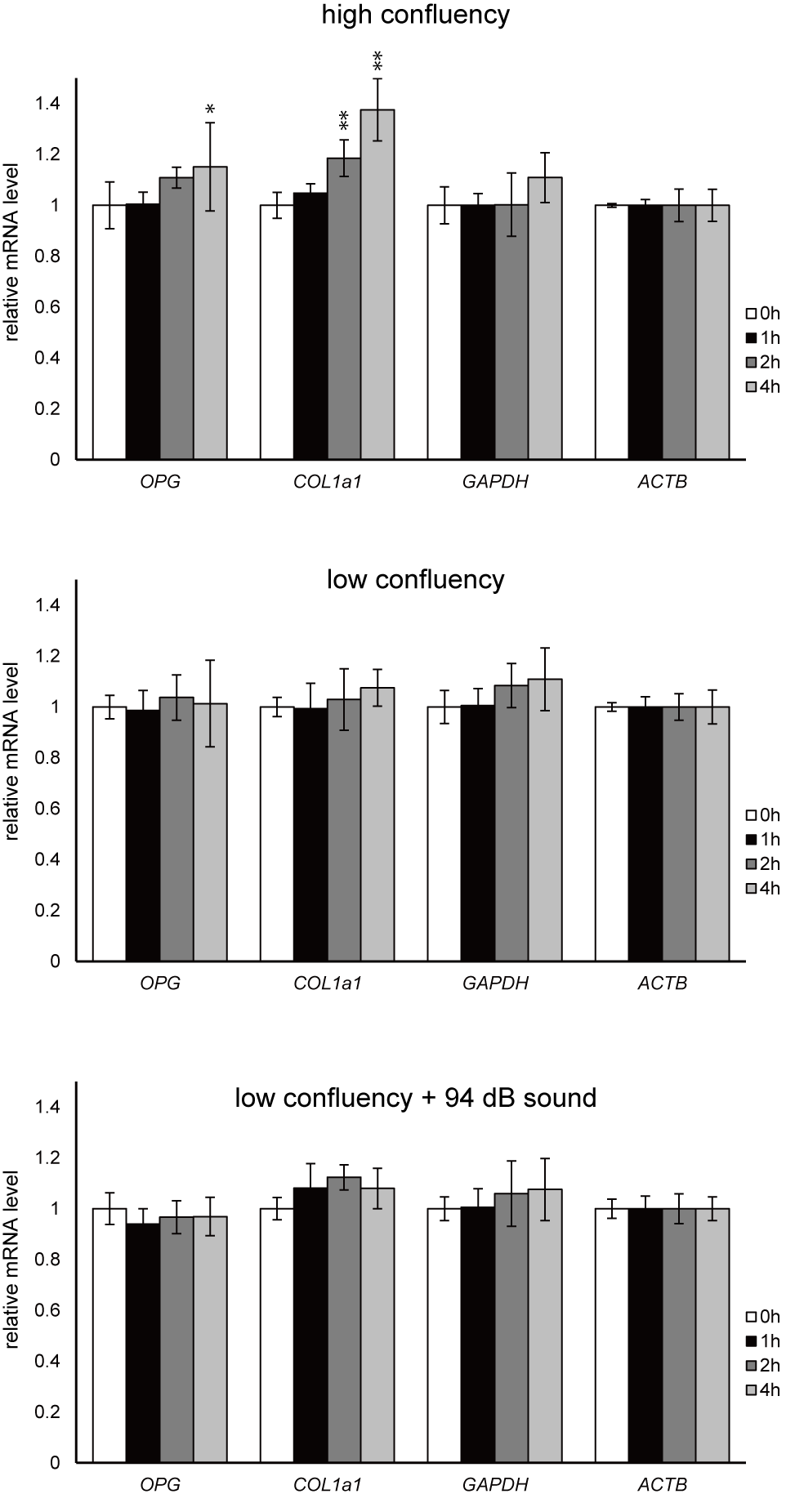

Supplement: S2 Fig — ST2 cells undergo spontaneous differentiation to osteoblast-like status in confluent culture conditions. High confluent culture spontaneously induces osteoblastic differentiation, which could be monitored by the significant increase in the OPG and COL1a1 mRNA levels (upper graph). This induction was not observed in low confluent status (middle graph). Sound emission did not change the expression levels of differentiation marker genes at low confluent status within 4 hours (lower graph). All the experiments in this study were performed below 60% confluence to exclude the effect of gene regulation accompanied by differentiation, by monitoring OPG or COL1a1 expression levels. (TIF) [file pone.0188764.s002.tif]
